# Clarendon Springs

**Published:** 1840

**Authors:** 


### CLARENDON SPRINGS, VERMONT.

Gentle reader in the west, do not be startled at the notice of a mineral water among the Green mountains. If you have a *sick* patient who wishes to escape from the heat and vapors of the southwest, to breathe an invigorating air, through the dog-days, why should he not go to Vermont, as well as New York or Virginia? Especially if he should find there, a different water from any to be found elsewhere in the United States? Mr. Hayes of Roxbury, Massachusetts, has analyzed this water, and finds it freer from *solid* ingredients, than common river water, but it abounds in *carbonic acid and nitrogen gases;* one gallon of the water containing 46-16 cubic inches of the former. It has therefore, a decidedly acidulous character; and closely resembles the SPA waters of Germany, which have long been celebrated in cutaneous affections. Dr. Gallup to whose pamphlet (1840,) we are indebted for our information, informs us that, little known as the Clarendon springs are, there is not wanting a considerable mass of evidence of their utility in cutaneous and urinary disorders; and that many of the cases cured by them, were from Mobile and other places in the south. This then may be one, and a new summer asylum for the invalids of the south; and we shall offer in the next paragraph, a compensating winter retreat for invalids of the North. D.

